# Role of gut microbiota in neuroinflammation: a focus on perioperative neurocognitive disorders

**DOI:** 10.3389/fcimb.2025.1582909

**Published:** 2025-07-07

**Authors:** Qun Zhou, Tuo Chen, Xiaoying Wang, Zhongnan Xu, Yue Song, Shan Liu, Yali Ge, Ju Gao

**Affiliations:** ^1^ Department of Anesthesiology, Northern Jiangsu People’s Hospital Affiliated to Yangzhou University/Clinical Medical College, Yangzhou University, Yangzhou, Jiangsu, China; ^2^ Department of General Surgery, Affiliated Hospital of Yangzhou University, Yangzhou University, Yangzhou, China

**Keywords:** perioperative neurocognitive disorders, gut microbiota, neuroinflammation, elder, gut-brain axis

## Abstract

Perioperative neurocognitive disorders (PND), including postoperative delirium, preoperatively cognitive impairment, postoperative cognitive dysfunction, and delayed neurocognitive recovery, represent common complications following anesthesia and surgery, especially in the elderly. As the global population ages, PND is receiving increasing attention due to its prolonged the hospitalization, reduced quality of life and elevated mortality rate. A growing body of evidence has been suggested that the gut-brain axis, a communication system between the gut microbiota and the neuroinflammation, plays a critical role in the development and progression of cognitive impairment. Perioperative interventions, including anesthesia, surgical stress, result in gut microbiota dysbiosis and subsequently trigger neuroinflammation. Therefore, it is necessary to clarify the underlying mechanistic associations between the gut microbiota and the neuroinflammation during PND progression. In this review, we synthesize current knowledge on the mechanistic interplay between gut microbiota dysbiosis and neuroinflammation in PND pathogenesis, which provide reasonable and novel therapeutic approaches targeting the gut-brain axis for PND.

## 1 Introduction

Perioperative neurocognitive disorders (PND) are common postoperative complications following anesthesia and surgery, encompassing conditions such as postoperative delirium, preoperative cognitive impairment, postoperative cognitive dysfunction, and delayed neurocognitive recovery (Dilmen et al., 2024; Evered et al., 2018). These disorders are often characterized by cognitive decline, including attention deficits, memory impairment, and reduced executive function, which may persist for months or even years after surgery (Rengel et al., 2023). Epidemiological studies indicate that the incidence of PND is approximately 23.8%, with rates as high as 50% in elderly cohorts (Silva et al., 2021; Travica et al., 2022). Furthermore, PND lead to prolonged hospitalization, reduced quality of life, and increased mortality rates (Decker et al., 2020). Although multiple risk factors for PND such as age, anesthetic management, surgery duration, postoperative pain, and infection have been identified, the underlying mechanisms remain unclear. Clinical study showed that preoperative dexamethasone administration reduced PND incidence by attenuating neuroinflammatory responses and enhancing stress resilience, highlighting the critical role of neuroinflammation in the pathogenesis of PND (Glumac et al., 2021). Therefore, investigating modulators of neuroinflammation may provide novel therapeutic avenues for PND management.

In recent years, the emerging role of gut microbiota in regulating central nervous system (CNS) function has attracted significant attention. Gut microbial homeostasis is essential for maintaining host health, including energy regulation, pathogen defense, immune system modulation, and gut barrier integrit (Lee et al., 2022; Asadi et al., 2022). Conversely, gut microbiota dysbiosis has been associated with various diseases, such as cardiovascular disorders, metabolic syndromes, and neuroinflammatory conditions (Iliodromiti et al., 2023; Hou et al., 2022). Although neurological diseases are traditionally considered as brain disorders, growing evidence suggest that their origins may lie in peripheral systems, particularly the gut microbiota. Recent researches have revealed that gut microbiota plays a pivotal role in regulating numerous neurological diseases, including depression, Parkinson’s disease, Alzheimer’s disease, traumatic brain injury, and PND, through the gut-brain axis (Wang and Kasper, 2014; Kasarello et al., 2023). In this article, we will review the mechanisms by which gut microbiota modulates neuroinflammation in PND and explore potential therapeutic strategies targeting the gut microbiota.

## 2 Neuroinflammation and PND

The administration of anesthesia and the trauma from surgery are well-known triggers for neuroinflammation, which contribute to the development of PND (Saxena and Maze, 2018; Yang et al., 2022). Exposure to general anesthesia alone has been shown to induce and exacerbate cognitive impairment, even absent surgical stress. This has been supported by substantial evidence from both clinical studies and animal models of PND. Recent clinical studies demonstrated that propofol, a commonly used intravenous anesthetic, significantly reduced hepatocyte growth factor and IFN-γ-induced IL-10 levels while increasing levels of IL-17, IL-5, and IL-7, indicating its pro-inflammatory effects on systemic inflammation (Qiao et al., 2015; Kallioinen et al., 2019). Similarly, studies in aged rats showed that sevoflurane and isoflurane contributed to PND by promoting the release of proinflammatory cytokines and activating microglia in the brain (Peng et al., 2012; Wang et al., 2018). Compared to anesthesia, surgery may cause more severe damage to cognitive function. Clinical studies revealed that surgery elevated postoperative concentrations of IL-6 and C-reactive protein levels, accompanied by activation of CNS inflammation, which correlated with cognitive impairment in patients (Liu et al., 2018; Neerland et al., 2016). A mouse model demonstrated that laparotomy, but not sevoflurane alone, induced neuroinflammation and tau phosphorylation (Huang et al., 2018). These findings suggest that the amplification of neuroinflammation following Anesthesia/surgery-triggered central inflammation play a significant role in the pathophysiology of PND. Hence, we focus on the neuroinflammation including microgliosis, astrogliosis and oligodendrocytes, and systematically review their function in PND.

### 2.1 Microglia

As the resident immunocompetent cells of the CNS, microglia derive from embryonic yolk sac progenitors and maintain continuous parenchymal surveillance through dynamic process motility (Masuda et al., 2022; Barry-Carroll and Gomez-Nicola, 2024). These neural macrophages execute essential synaptic regulatory functions including activity-dependent pruning and circuit refinement, thereby preserving neuroplastic homeostasis (Chowen and Garcia-Segura, 2020). While the role of microglia in neurological conditions is complex, their activation can lead to neuroinflammation in rodent models of PND following peripheral surgery and are associated with long-lasting cognitive impairments in humans (Lin et al., 2017; Forsberg et al., 2017). Accumulating evidence indicated that anesthesia and surgery caused varying degrees of microglial activation, triggering an inflammatory cascade that promoted the synthesis and secretion of inflammatory mediators such as cytokines, eicosanoids, complement factors, excitatory amino acids, reactive oxygen species, and nitric oxide (Hanisch, 2002; Minagar et al., 2002). *In vivo*, depletion of microglia induced by colony-stimulating factor 1 receptor inhibitors reduced neuroinflammation, as evidenced by decreased hippocampal levels of inflammatory cytokines, reduced microglial activation, and decreased recruitment of leukocytes in PND mouse models (Henry et al., 2020). *In vitro*, lipopolysaccharide (LPS) induced oxidative stress and promoted the release of inflammatory cytokines in microglia-like cell lines (BV-2 cells) (Butturini et al., 2019). Consequently, targeting microglia through depletion or inhibition of their activation have emerged as a promising therapeutic strategy for treating PND.

### 2.2 Astrocytes

Astrocytes, the most abundant glial cells in the CNS, play a crucial role in maintaining metabolic balance, recycling neurotransmitters, providing neurons with essential nutrients, and participating in immune responses (Lee et al., 2022). Astrocyte activation is a key contributor to PND in models of major surgeries, such as fracture surgery, orthopedic surgery, and neural surgery (Wan et al., 2010; Xiong et al., 2018; Femenía et al., 2018). Upon stimulation, astrocytes undergo significant morphological, transcriptional, and functional changes, transitioning into reactive states characterized by either neurotoxic (A1) or neuroprotective (A2) phenotypes. Systemic inflammatory mediators during the perioperative period can invade the brain and promote the neurotoxic A1 astrocyte phenotype, releasing a broad range of pro-inflammatory mediators that exacerbate neuroinflammation. A1 astrocytes, often activated by signals from microglia and systemic inflammation, promote neuronal death and contribute to PND progression (Li et al., 2020; Franklin et al., 2021). In contrast, A2 astrocytes support cell survival and exert protective effects, highlighting the dual roles astrocytes play in CNS pathology (Fan and Huo, 2021). Therefore, maintaining the balance between A1 and A2 astrocytes to suppress neuroinflammation is a crucial strategy for treating PND.

### 2.3 Oligodendrocytes

Oligodendrocytes, crucial glial components in the CNS, are essential for axonal myelination and neuronal maintenance (Allen and Lyons, 2018; Ortinski et al., 2022). Research has shown that anesthesia can directly act on surface receptors of oligodendrocytes and induce neuroinflammation, impairing their proliferation, differentiation, and survival, ultimately contributing to the onset and progression of PND. Clinical studies indicated that anesthesia in children undergoing surgery led to oligodendrocyte apoptosis during myelination, resulting in decreased white matter integrity and volume, thereby increasing the risk of cognitive impairments and structural brain changes (Block et al., 2017; Banerjee et al., 2019). *In vivo* study demonstrated that propofol selectively affected oligodendrocytes, causing apoptosis in both neurons and oligodendrocytes in the brains of fetal and neonatal rhesus monkeys after 5 hours of exposure (Creeley et al., 2013). Moreover, repeated exposure to sevoflurane activate microglia and systemic inflammation in the brains of mice, leading to oligodendrocyte damage, which play a key role in the development of PND (Shen et al., 2013; Xia et al., 2017). Therefore, therapeutic strategies targeting neuroinflammation mediated by oligodendrocyte dysfunction represent a promising treatment for PND.

## 3 Gut microbiota and PND

As the most complex and densely populated ecosystem in the human body, the gut harbors a diverse and dynamic community of microorganisms, collectively known as the gut microbiota. The gut microbiota, consisting of bacteria, viruses, archaea, protists, and fungi, is estimated to consist of approximately 10^13^ -10^14^ microorganisms, roughly 10 times the number of human cells in the body, and its collective genetic material surpasses the human genome by more than 100 times (Thursby and Juge, 2017; Zhang et al., 2023; Aya et al., 2021). In recent years, gut microbiota has gained significant attention, highlighting the intricate connection between microbial composition and human health. Emerging evidence reveal that the gut microbiota plays a crucial role in regulating immune homeostasis, maintaining mental health, modulating brain function, behavior, and metabolism (Tang et al., 2017; Krezalek and Alverdy, 2023; Dinan and Cryan, 2017; Morais et al., 2021). However, gut microbiota dysbiosis has been associated with a wide range of neurological and neuropsychiatric disorders, including Alzheimer’s disease, Parkinson’s disease, and other cognitive impairments (Vogt et al., 2017; Sochocka et al., 2019; Zhan et al., 2018; Sampson et al., 2016). Meanwhile, the emerging roles of gut microbiota in the PND have been attracting more attention. In animal models, alterations in the gut microbiome have been shown to induce neuroinflammation, oxidative stress, and changes in neurotransmitter levels, all of which contribute to cognitive impairment (Clarke et al., 2013). Studies using germ-free (GF) mice have provided strong evidence for the critical role of gut microbiota in PND. GF mice exhibited abnormal brain function, manifesting as learning disabilities, anxiety-like behavior, and reduced social skills. Furthermore, GF mice transplanted with fecal microbiota from PND patients displayed impaired learning and memory (Kundu et al., 2022; Luczynski et al., 2016). Clinical studies also demonstrated that surgical/anesthesia disrupted gut microbiota, leading to dysbiosis characterized by age-dependent reductions in microbial abundance and diversity during the perioperative period (Guyton and Alverdy, 2017; Liufu et al., 2020). Collectively, these findings have significantly advanced our understanding of the gut-brain axis. Therefore, exploring the role of perioperative gut microbiota and elucidating the gut-brain axis will enhance our understanding of the relationship between gut microbiota and PND.

### 3.1 Characteristics of gut microbiome in the perioperative period

The perioperative period encompasses the preoperative, intraoperative, and postoperative phases, focusing on patient preparation, surgical and anesthetic management, and postoperative recovery. During the preoperative phase, patients commonly undergo gastrointestinal preparation, adhere to dietary and fluid restrictions, and experience psychological stress associated with surgery, all of which can significantly impact the gut microbiota. Recent studies indicated that mechanical bowel preparation before surgery increased the risk of PND by altering the gut microbiota, with these changes taking up to 14 days for the majority of the intestinal microbiota to return to baseline composition (Yang et al., 2022; Nagata et al., 2019). Preoperative fasting and fluid restriction are standard practices to reduce the risk of aspiration and other complications. However, emerging evidence highlight that diet play a critical role in shaping the community structure and functionality of gut microbiota (Carmody et al., 2015; Beam et al., 2021). Unlike intermittent fasting, a health-focused dietary strategy involving timed eating cycles, prolonged fasting is primarily utilized as a perioperative dietary protocol to optimize surgical outcomes. However, prolonged fasting or dietary modifications before surgery can disrupt the gut microbial balance, potentially leading to significant alterations in microbial diversity and metabolic activity. These changes may weaken immune function, increase susceptibility to infections, and alter inflammatory responses (Cantoni et al., 2022; Wang et al., 2024). Animal studies showed that fasting made intestinal tissues highly susceptible to nutrient deprivation, impairing mucosal immunity and weakening the immune barrier function, which could lead to immune dysfunction and excessive hypersensitivity reactions (Nagai et al., 2019; Okada et al., 2013). Surgical patients are also subjected to various forms of stress during the preoperative period, including psychological stress, as well as physiological stress (Kohl et al., 2014). These stressors can activate the sympathetic nervous system and the hypothalamic-pituitary-adrenal (HPA) axis, resulting in imbalances of gut microbiota, increasing intestinal permeability and heightening inflammation. Research indicated that psychological stress was documented to impair the colonization of microorganisms on mucosal surfaces, thereby weakening the protective barrier of the gut and increasing the host susceptibility to infections (Wei et al., 2024; Vanuytsel et al., 2014). Notably, the susceptibility of elderly patients to PND is closely associated with gut microbiota. Compared to younger individuals, elderly patients typically exhibit reduced gut microbiota diversity, with a decline in beneficial bacterial groups such as *Firmicutes* and *Actinobacteria* and an increase in pathogenic groups such as *Proteobacteria* (Rondanelli et al., 2015; McLeod et al., 2023). Additionally, serum levels of short-chain fatty acids (SCFAs), vital bacterial metabolites produced in the colon, are lower in elderly individuals (Sanna et al., 2019). These factors contribute to the vulnerability of elderly patients during the perioperative period and may affect their overall recovery and outcomes.

During the intraoperative phase, the primary factors contributing to PND are surgical and anesthetic management. Previous studies suggested that the high risk of PND related to anesthesia was primarily contributing to neurotransmitter imbalances and the direct impact of anesthetic agents on the neural network of brain (Liu et al., 2022). However, evidence from animal models indicated that anesthesia was associated with gut microbiota dysbiosis, disrupting the production of essential endocrine and metabolic products by the microbiota (Xu et al., 2020; Serbanescu et al., 2019). Furthermore, studies conducted in experimental animal models demonstrated that surgery was an independent factor in the occurrence of PND (Zhang et al., 2019; Lian et al., 2021). Surgical stress-induced gut microbiota dysbiosis in mice has been related to dysfunctions in multiple neurotransmitter systems, particularly the serotonin [5-hydroxytryptamine (5-HT)] and gamma-aminobutyric acid (GABA) systems (Lu et al., 2022). Clinical studies have shown that surgical interventions, particularly abdominal surgery, significantly impact the gut microbiota. Abdominal surgery often involves intestinal rerouting and anastomosis, such as end-to-end, end-to-side, and side-to-side anastomosis, to restore digestive function or treat disease by reconstructing or bypassing diseased intestinal segments while directly altering the gut microbiota environment (Huang et al., 2023). Research found that the diversity and abundance of gut microbiota increased in patients with gastric cancer undergoing surgery (Erawijantari et al., 2020). Additionally, gastrectomy lead to an increase in the abundance of aerobic bacteria, facultative anaerobic bacteria adapted to aerobic and anaerobic environments, and oral microbes, which may be associated with digestive tract reconstruction and postoperative complications. Interestingly, although mice exhibiting delirium-like behavior after general anesthesia and surgical laparotomy showed distinct gut microbiota dysbiosis, transplanting gut microbiota from mice without delirium to those exhibiting delirium-like behaviors improved cognitive symptoms (Zhang et al., 2019).

During the postoperative period, factors such as infection and pain significantly influence PND. The gut microbiota plays a dual role in maintaining host defense by functioning as a biological barrier that inhibits pathogen colonization through competitive exclusion and by modulating the host immune response to efficiently identify and eliminate invading microorganisms (Schmitt et al., 2019). Surgery induces gut microbiota dysbiosis, gut barrier damage, and intestinal inflammation, contributing to postoperative infection. A pilot study involving 26 renal transplant patients revealed significant alterations in gut microbiota within fecal samples collected three months post-surgery, which were associated with complications including diarrhea, acute rejection, and urinary tract infections (Lee et al., 2014). A systematic review highlighted that alterations in gut microbiota following gastrointestinal surgery might contribute to the onset of postoperative infection such as anastomotic leakage and wound infection. These alterations are characterized by an increase in potential pathogens such as *Pseudomonas*, *Staphylococcus*, and *Enterococcus*, alongside a reduction in beneficial microbes like *Lactobacillus* and *Bifidobacterium* (Lederer et al., 2017). Although antibiotics were essential for preventing postoperative infections, numerous studies demonstrated that antibiotics could have both short- and long-term impacts on the gut microbiota in humans and animals, including alterations in microbiota composition, reduced diversity, and delays in microbial colonization (Francino, 2015). Antibiotics can elevate the risk of postoperative infections by disturbing the gut microbiota and its associated metabolic processes, thereby disrupting the natural defense mechanisms and enhancing susceptibility to subsequent infections (Kim, 2015). Additionally, postoperative pain can lead to gut microbiota dysbiosis, as the stress and inflammatory responses triggered by pain may disrupt the delicate balance of gut microbiota (Pusceddu and Gareau, 2018). Moreover, the use of analgesic medications, such as opioids, can exacerbate this dysbiosis, damage the intestinal epithelial barrier function and promote the translocation of gut microbiota, which increased susceptibility to PND (Mora et al., 2012; Meng et al., 2013). Recent studies in both human and animal models, summarized in **Table 1**, further provide evidence of the relationship between gut microbiota and PND.

**Table 1:** Outlining the studies focused on the relationship between the gut microbiota and PND.

Type of study	Reference	Setting	Participants	Main findings on the gut microbiota
Observation	(Liu et al., 2022a)	Elderly patients undergoing abdominal surgery	40 patients over 65 years of age (age mean 71.35)	The characteristics of gut microbiota in PND patients before surgery included higher abundance of *Proteobacteria, Enterbacteriaceae, E. shigella, Klebsiella, Ruminococcus, Roseburia, Blautia, Holdemanella, Anaerostipes, Burkholderiaceae, Peptococcus, Lactobacillus*, and *Dorea, and* lower abundance of *S. equinus* and *B. hominis*. *E. shigella*, as a promising diagnostic bacterial species, could predict PND.
Intervention, (MBP *vs* no MBP)	(Yang et al., 2022)	Elderly patients undergoing gastric cancer surgery	81 patients over 65 years of age (age mean 73)	MBP was related with the higher risk of PND and led to changes in gut microbiota, including higher abundance of *Bacteroides* and *Veillonella*, and lower abundance of *Olsenella*.
Observation	(Kim et al., 2023)	Elderly korean patients	80 patients aged 57–80 years (age mean 69.5)	The gut microbiota of cognitive impairment patients were mainly characterized by increased *Bacteroides* and decreased *Prevotella*, and *Akkermansia.*
Observation	(McLeod et al., 2023)	Elderly African American patients	60 patients aged 55–76 years	The gut microbiota were associated with cognitive impairment, which included increased *Str eptococcus*, *Ruminococcaceae UCG-002*, *Methanobrevibacter*, *Bifidobacterium*, *Dialister invisus*, and decreased *Parabacteroides distasonis*
Observation	(Maekawa et al., 2020)	Patients undergoing cardiac surgery	21 patients aged 22- 80 years (age median 62)	*Staphylococcus* and *Pseudomonas* counts were significantly increased in delirium patients after cardiac surgery, and positively correlated with pseudopsia and delirium.
Observation	(Zhang et al., 2023)	Patients undergoing orthopedic surgery	86 patients (median age 71)	*Parabacteroides distasonis* from postoperative fecal samples was positively related to delirium.
Mendelian randomization	(Yu et al., 2023)	GWAS data for gut microbiota and delirium	18,340 participants from 24 cohorts	The presence of *Desulfovibrionaceae*, *Desulfovibrionales*, and *Candidatus Soleaferrea* was associated with increased delirium risk, while *Oxalobacteraceae*, *Holdemania*, *Ruminococcus gnavus*, and *Eggerthella* were linked to a reduced risk.
Observation	(Zhang et al., 2023)	Anesthesia/surgery-induced delirium model	The aged male C57BL/6 mice	Anesthesia and surgery in aged mice induced cognitive impairment and depression, potentially mediated by alterations in gut microbiota, including reduced levels of beneficial bacteria like *Lachnospiraceae*, *Butyrivibrio*, and *Eubacterium*, as well as metabolites such as long-chain unsaturated fatty acids, spermidine, and choline, which are crucial for brain function and neurotransmitter synthesis.
Observation	(Zhang et al., 2019)	Abdominal surgery-induced delirium model	The male C57BL/6 mice	Gut microbiota dysbiosis, characterized by reduced diversity, increased pathogenic bacteria such as *Gammaproteobacteria* and *Escherichia-Shigella*, and decreased beneficial bacteria like *Bifidobacteriales*, *Ruminococcaceae*, and *Butyricimonas*, plays a crucial role in the pathogenesis of PND
Observation	(Lian et al., 2021)	Anesthesia/surgery-induced delirium model	The aged male C57BL/6 mice	Anesthesia/surgery in aged mice led to behavioral changes, gut microbiota dysbiosis with increased levels of *Fusobacteria*, *Proteobacteria*, and *Escherichia–Shigella*, and altered metabolites such as tryptophan and kynurenine, which together might contribute to the development of PND.
Observation	(Pan et al., 2023)	Surgery-induced delirium model	The aged male C57BL/6 mice	Gut microbiota dysbiosis induced by surgery increased intestinal permeability and alters key microbial metabolites, which promoted neuroinflammation. Moreover, fecal microbiota transplantation from young mice restored cognitive function and reduced inflammation in aged mice, highlighting the potential for therapeutic strategies targeting gut microbiota to prevent PND.
Intervention (probiotic VSL#3 or mixed antibiotics *vs* water)	(Jiang et al., 2019)	Abdominal surgery-induced delirium model	The aged male C57BL/6 mice	Pretreatment with probiotics (VSL#3) or mixed antibiotics could prevent PND via gut microbiota–brain axis.
Intervention (B-GOS supplementation *vs* water)	(Yang et al., 2018)	Surgery-induced delirium model	Adult rats	B-GOS, a prebiotic, alleviated surgery-induced PND and neuroinflammation in rats by modulating the gut microbiota and inhibiting the activation of harmful M1 microglia.
Intervention (*Lactobacillus vs* water)	(Liufu et al., 2020)	Anesthesia/surgery induced delirium model	Female mice	Treatment with *Lactobacillus* could improve anesthesia/surgery induced age-dependent behavioral changes, gut microbiota dysbiosis, brain IL-6 levels, synaptic markers, and mitochondrial dysfunction.
Intervention *Lactobacillus* or sodium butyrate *vs* water)	(Wen et al., 2020)	Anesthesia/surgery-induced delirium model	The male C57BL/6 mice	*Lactobacillus* or sodium butyrate ameliorate Anesthesia/surgery-induced PND via restoring blood-brain barrier.

### 3.2 Gut-brain axis

Recent studies have identified a strong connection between gut microbiota and PND through the gut-brain axis. The gut-brain axis refers to the bidirectional communication between the gut and the brain, traditionally understood to involve the integration of immunological, neural, and hormonal signals (Russo et al., 2018). However, recent evidence highlight the gut microbiota as a critical gastrointestinal factor that significantly influence and modulate this axis (Zhang et al., 2023). Historically, research on the gut-brain axis primarily focused on its role in functional gastrointestinal disorders, such as irritable bowel syndrome and inflammatory bowel disease (Rea et al., 2017). However, dysregulation of this axis has been implicated in the pathogenesis of neurological disorders (Russo et al., 2018; Wang et al., 2021; Heiss and Olofsson, 2019). The gut-brain axis involved in the mechanism of PND comprises multiple communication routes, including gut barrier and the blood-brain barrier (BBB), endocrine, neural pathways, the immune system and gut microbiota metabolism.

The barriers located within the gut and brain are specialized cellular structures essential for maintaining the strict homeostasis of distinct compartments along the gut-brain axis. These include the gut barrier and the BBB, which serve as key gateways for communication between the gut, microbiota, and brain. Mounting evidence showed that compromised gut barrier and BBB dysfunction were relevant to a wide range of cognitive disorders (Pellegrini et al., 2023; Xu et al., 2020; Morton et al., 2023). When the host experiences stress or infection, gut microbiota composition changes, potentially disrupting gut and brain barrier function. An impaired gut barrier allow abnormal translocation of microbial metabolites and structural components into the bloodstream, which may then reach the brain barriers (Leigh et al., 2023). Furthermore, the uncontrolled translocation of these microbial components can trigger an inflammatory response, resulting in neuroinflammation and subsequent cognitive dysfunction (Spadoni et al., 2017).

The gut-brain axis is intricately regulated by neural pathways, particularly the vagus nerve, which transmits sensory information from the gut to the brain (Tan et al., 2022). In the perioperative period, surgical stress and anesthesia can disrupt vagal tone, impairing gut-brain communication and potentially affecting the response of brain to external stimuli, mood regulation, and cognitive functions (Gacias et al., 2016). Additionally, the enteric nervous system communicates with the brain through signals generated by enteric neurons, which are influenced by changes in the gut luminal environment shaped by the microbiota. Altered enteric signaling may disrupt CNS function and contribute to cognitive decline following surgery (Cryan et al., 2019). Furthermore, various neurotransmitters are essential for the bidirectional signaling between the gut and the brain. Notably, abnormal neurotransmission can disrupt the HPA axis, enteric nervous system, and alter the gut microbiota, leading to neuronal damage and cognitive impairments (Grinevich et al., 2001; Farzi et al., 2018).

**Figure 1:**
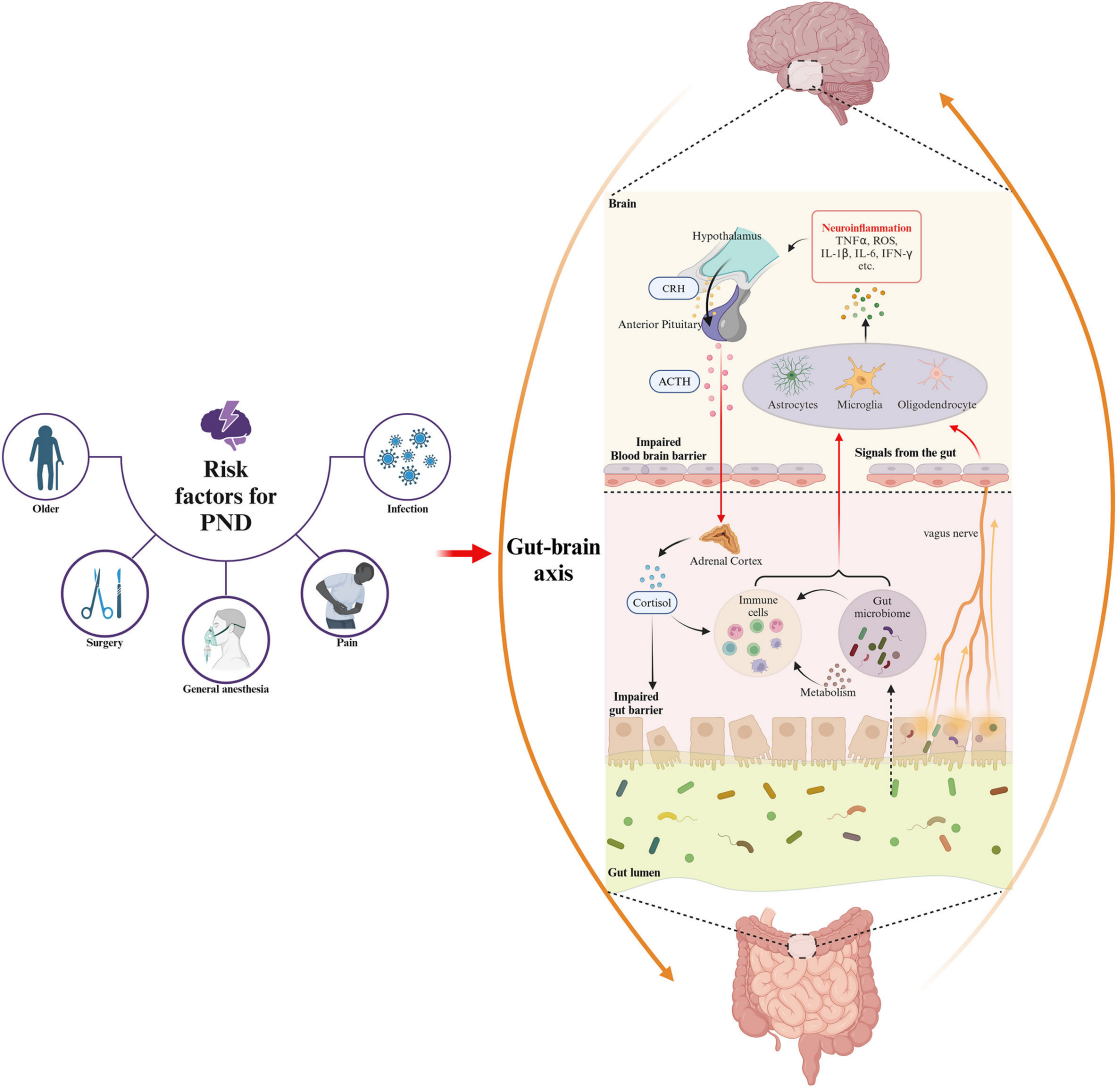
The risk factors for PND in the perioperative period and gut microbiota involvement in the underlying mechanisms of PND based on gut-brain axis. Perioperative risk factors for PND, including older, anesthesia, surgical stress, prolonged fasting, and use of antibiotic or analgesic, disrupt gut microbiota composition and diversity, thereby inducing dysbiosis. This dysbiosis exacerbates neuroinflammation through gut-brain axis pathways involving gut barrier and BBB damage, endocrine dysfunction, neural disruption, immune activation, and microbial metabolite alteration.

An imbalance in microbial metabolism resulting from dysbiosis has been suggested to disrupt cognitive functions, potentially contributing to the development of PND (Zhao et al., 2019). The gut microbiota synthesizes neurotransmitters such as 5-HT and GABA, which can interact directly with the nervous system or modulate the host neurotransmitter production, thereby influencing neurological processes (Deczkowska et al., 2018). Besides, the gut microbiota produces a diverse range of metabolites including SCFAs, such as acetate, propionate, and butyrate. These metabolites can cross the barrier system and modulate the activity of glial cells and neurons, influencing processes such as neuroinflammation, neurogenesis, and neurotransmission (Erny et al., 2015). The gut microbiota significantly influences the systemic immune system, and dysbiosis can facilitate the translocation of bacterial components into the bloodstream, amplifying systemic inflammation and impacting brain function (Deczkowska et al., 2018). Furthermore, gut microbiota components, such as LPS, can cross the gut barrier, initiating immune responses and resulting in an intensified inflammatory state. Inflammatory cytokines, including IFN-γ, IL-1β, IL-6, and TNF-α, along with other mediators, can reach the CNS, contributing to neuroinflammation, a key factor in the development of PND (Erny et al., 2015). The perioperative risk factors for PND and its impact on the brain-gut axis are summarized in **Figure 1**.

## 4 Role of gut microbiota in neuroinflammation based on gut-brain axis

**Figure 2:**
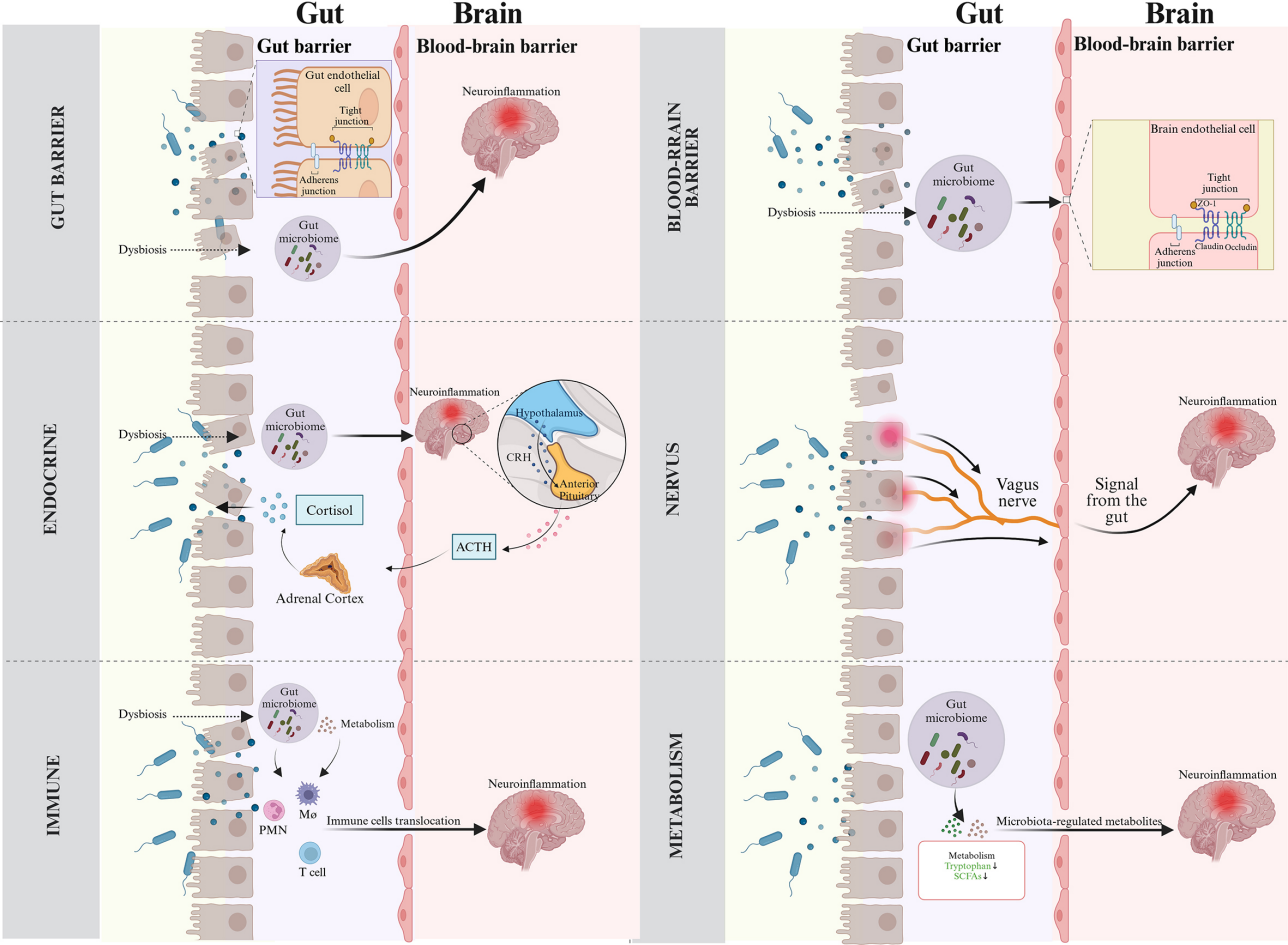
Routes of gut microbiota for neuroinflammation across the gut-brain axis. (Top left) Gut barrier. Disruption of gut barrier induced by microbial dysbiosis facilitates the translocation of bacteria and their metabolites into bloodstream, triggering immune and inflammatory responses that can exacerbate neuroinflammation. (Top right) Blood-brain barrier. Gut-derived inflammatory factors can damage BBB and lead to neuroinflammation. (Middle left) Endocrine system. Gut microbiota results in the disorders of the endocrine system, mainly involving HPA axis activation, which eventually leads to neuroinflammation. In addition, inflammation-induced activity of the HPA axis triggers systemic release of cortisol, which modifies gut barrier function. (Middle right) Neural pathways. Microbiota-mediated inflammatory signals utilize the vagus nerve as a high-speed conduit to reach the brain, triggering neuroinflammatory responses. (Bottom left) Immunity. Gut microbiota stimulates inflammatory immune cells, which then migrate to the CNS and aggravate neuroinflammation. (Bottom right) Gut microbiota metabolites. Metabolites from the gut microbiota guide the development and neuroinflammatory responses.

Neuroinflammation refers to the activation of immune responses in the CNS, involving immune cells such as microglia and astrocytes (Yang et al., 2023). While neuroinflammation is beneficial in maintaining brain homeostasis and defending against infection or injury, it becomes chronic and dysregulated in CNS diseases like PND, leading to neuronal damage and cognitive impairment (Cheng et al., 2022; Mou et al., 2022). Accumulating evidence highlight that the gut microbiota plays significant role in neuroinflammation onset and progression via the gut-brain axis, a complex network enabling bidirectional communication between the gut microbiota and the CNS. This axis involves multiple pathways, including gut barrier and the BBB, endocrine, neural pathways, the immune system and gut microbiota metabolism (Wang and Wang, 2016). In the following section, we will explain how the gut microbiota induces neuroinflammation through these pathways based on current research (**Figure 2**).

### 4.1 Gut barrier

The gut barrier, composed of the mucus layer, epithelial barrier, and vascular barrier, serves as a multilayered defense system that protect the host from external threats and regulate essential intestinal functions (Chelakkot et al., 2018). The mucus layer, primarily consisting of water and mucins secreted by goblet cells, plays a critical role in maintaining gut homeostasis by producing bacteriostatic peptides and maintaining a physical barrier between gut microbiota and the mucosa (Gustafsson and Johansson, 2022). This layer not only influences gut microbiota composition, nutrient absorption, and secretory functions but also modulates intestinal permeability and immune responses (Breugelmans et al., 2022). The activity of goblet cells is modulated by the gut microbiota and its metabolites, particularly SCFAs. Previous studies found that butyrate and acetate enhanced mucus production, increase its thickness, and upregulate MUC-2 expression through the release of prostaglandin E1. Additionally, butyrate contributes to the maintenance of epithelial barrier integrity and host immunity (Willemsen et al., 2003; Kelly et al., 2015). The epithelial barrier, located beneath the mucus layer, is a single layer of epithelial cells organized into villi and crypts. These cells, including enteroendocrine cells, tuft cells, Paneth cells, stem cells, and enterocytes, perform specialized functions such as hormone secretion, antimicrobial peptide production, lipid absorption, and immunoglobulin secretion (Peterson and Artis, 2014). The cells of the intestinal epithelial barrier are tightly bound together by junctional complexes, a dynamic structure composed of transmembrane proteins, including tight junctions, adherens junctions, gap junctions, and desmosomes, which maintain the intestinal epithelial barrier integrity and regulate the paracellular transport of solutes and fluids (Odenwald and Turner, 2017). Gut microbiota and their metabolites regulate the permeability of intestinal tight junctions and epithelial barrier function by activating pattern recognition receptors and intracellular signaling pathways in epithelial cells (Abraham et al., 2022). The vascular barrier, the innermost layer of the gut barrier, controls the passage of intestinal contents into the bloodstream. Composed of endothelial cells connected by tight and adherens junctions, along with supporting cells such as enteric glial cells, pericytes, and fibroblasts, this barrier prevents the translocation of bacteria, toxins, and inflammatory mediators into systemic circulation (Spadoni et al., 2015). Under normal conditions, the gut microbiota and the gut barrier maintain immune homeostasis by distinguishing between commensal bacteria and pathogens, thereby regulating PRR expression and preventing excessive inflammation (Artis, 2008; Belkaid and Hand, 2014). However, disruptions in gut microbiota balance, caused by factors such as infection, trauma, surgery, or anesthesia, can compromise the gut barrier. These result in increased translocation of bacteria and their byproducts into systemic circulation, amplifying mucosal and systemic inflammation, including neuroinflammation. Moreover, recent studies highlighted the role of postoperative gut dysbiosis in cognitive dysfunction, linking it to impaired gut barrier function, metabolic abnormalities, and neuroinflammation (Pan et al., 2023). In summary, the gut barrier is a dynamic and multifaceted system that serves as the first line of defense against external threats. Disruption of this barrier, often resulting from microbial dysbiosis, facilitates the translocation of bacteria and their metabolites into the mucosa and bloodstream, triggering immune and inflammatory responses that can propagate to the brain and exacerbate neuroinflammation.

### 4.2 BBB

BBB is a critical interface connecting the gut microbiota and neuroinflammation. The BBB is a dynamic, highly selective semipermeable membrane that serves as a protective filter, preventing harmful toxins and pathogens from entering the brain while allowing essential nutrients and oxygen to pass through. It is composed of endothelial cells tightly bound by tight junctions and adherens junctions, which maintain the integrity of barrier. The tight junctions are formed by transmembrane proteins such as claudins, occludins, and JAMs, which are linked to cytosolic adapter proteins like ZO-1 and ZO-2, providing structural connections to the cytoskeleton (Kadry et al., 2020).

Numerous studies have shown that BBB disruption is closely associated with the onset or progression of neuroinflammation (O’Keeffe et al., 2020; Bowman et al., 2018).Changes in specific microbial communities can compromise BBB integrity, as observed in patients with anti-NMDA receptor encephalitis, where increased BBB permeability allows harmful substances to enter the brain, triggering neuroinflammation (Gong et al., 2024). Moreover, excessive activation of glial cells and inflammation exacerbate BBB damage, intensifying neuroinflammation (Takata et al., 2021). On the other hand, studies suggest that prebiotics and probiotics can influence the gut microbiota to reduce colonic inflammation, preserve both gut and BBB integrity, prevent the activation of microglial inflammatory phenotypes, and decrease inflammatory cytokine levels in the bloodstream and hippocampus (Yang et al., 2018). Therefore, the gut microbiota impacts BBB integrity and function, thereby regulating neuroinflammation.

Recent studies have demonstrated that the gut microbiota significantly impacts BBB function. Antibiotic-treated mice and GF mice exhibit increased BBB permeability and dysregulated endothelial tight junctions, while fecal transplantation from normal mice to GF mice reduces BBB permeability and restores tight junction integrity (Braniste et al., 2014). Additionally, long-term high-fat diet-induced gut microbiota dysbiosis has been shown to cause BBB disruption, leading to cognitive decline (Thériault et al., 2016). Certain gut microbiota metabolites, such as SCFAs, enhance BBB function by regulating the actin cytoskeleton dynamics and its interaction with tight junction proteins (Knox et al., 2022). SCFAs also reduce inflammation by activating G-protein-coupled receptors and inhibiting NF-κB signaling, thereby further enhancing BBB integrity (Liu et al., 2020). Conversely, LPS disrupts the BBB by directly damaging endothelial cells, activating TLR4 to trigger NF-κB expression and systemic inflammation, which increases tight junction permeability, induces pericyte degeneration and endothelial apoptosis, and promotes astrocyte foot process phagocytosis (Zhao and Lukiw, 2018; Haruwaka et al., 2019).

### 4.3 The HPA axis

The HPA axis plays a crucial role in the gut-brain axis, contributing to the regulation of stress responses and various physiological processes, including digestion, immune function, mood and emotions, sexual behavior, and energy balance (Leistner and Menke, 2020). It is a neuroendocrine system that mediates response to both internal and external stressors. Under normal conditions, the HPA axis activity displays ongoing oscillations that are synchronized with both circadian and ultradian rhythms (Androulakis, 2021; Focke and Iremonger, 2020). When activated, the HPA axis stimulates neurons in the paraventricular nucleus of the hypothalamus to release corticotropin-releasing hormone (CRH). This, in turn, prompts the anterior pituitary to secrete adrenocorticotropic hormone (ACTH), which ultimately triggers the release of cortisol from the adrenal cortex.

Growing evidence highlight the importance of gut microbiota and its signals in the proper development of the HPA axis. Recent studies found that GF mice exhibited an exaggerated HPA axis response to restraint stress (Clarke et al., 2013; Crumeyrolle-Arias et al., 2014). Notably, the exaggerated HPA response in GF mice can be partially normalized by colonizing them with fecal samples from wild type mice (Sudo et al., 2004). Additionally, probiotic supplementation can alleviate stress-induced HPA axis dysfunction, reduce neuroinflammation, and improve cognitive impairment (Liang et al., 2015). Clinical studies have shown that gut microbiota diversity from1-month-old infants were related to stronger HPA axis reactivity. Specifically, higher relative abundances of *Bifidobacterium* and *Streptococcus*, along with a lower relative abundance of *Bacteroides* in early infancy, were associated with enhanced cortisol reactivity (Rosin et al., 2021). Another study showed that gut microbiota diversity influenced the salivary cortisol stress response in 2.5-month-old children (Keskitalo et al., 2021). Normal cortisol secretion help suppress peripheral cytokine production, thereby mitigating inflammation (Tofani et al., 2025; Jahnke et al., 2021). However, chronic excessive cortisol secretion due to HPA axis hyperactivity promotes inflammation by downregulating glucocorticoid receptors, which are typically involved in anti-inflammatory responses. In this context, elevated levels of proinflammatory cytokines, such as IL-1β and TNF-α, drive excessive activation of both peripheral and central immune systems. This overactivation leads to the priming and trafficking of monocytes, which in turn alters the phenotype of microglia and ultimately triggers neuroinflammation (Knezevic et al., 2023; Wohleb et al., 2014). Furthermore, studies have shown that HPA axis dysfunction, whether characterized by cortisol hypersecretion, is associated with the onset of neuroinflammation of PND following surgery (Galvis et al., 2022; Rasmussen et al., 2005). Collectively, the HPA axis acts as a pivotal role in the gut-brain axis by integrating gut microbiota and neuroinflammation, which highlight its potential therapy for PND.

### 4.4 Vagus nerve

The gut and brain are directly connected through bidirectional neural pathways, with the vagus nerve serving as the primary route (Asadi et al., 2022; Breit et al., 2018). This nerve originates from the brainstem and extensively innervates multiple organs, including the gastrointestinal tract (Brookes et al., 2013). Vagal afferent fibers project from terminal endings within the intestinal wall, forming specialized sensory receptors that detect chemical, thermal, osmotic, and mechanical stimuli (Berthoud and Neuhuber, 2000).

As a critical component of the gut-brain axis, the vagus nerve acts as a high-speed conduit for transmitting microbiota-mediated inflammatory signals to the brain, thereby initiating neuroinflammatory responses (Powley et al., 2019). Under physiological conditions, microbial-derived signaling molecules bind to receptors on vagal fibers, transmitting homeostatic information to the brain. However, pathological stressors such as surgery, infection, trauma, or chronic stress can disrupt this equilibrium, promoting overgrowth of pathogenic species or depletion of beneficial microbiota. Such dysbiosis may activate pro-inflammatory cascades, resulting in immune dysregulation and neuroinflammation (Campbell et al., 2023). Mechanistically, peripherally elevated glutamate induced by inflammation binds to NR2B, a subtype of NMDA receptor, on brain mast cells via vagal afferents, triggering release of pro-inflammatory mediators, BBB disruption, and subsequent neuroinflammation (Yang et al., 2022). Supporting this pathway, fecal samples from stress-induced depression models to healthy mice induces hippocampal neurogenesis deficits and neuroinflammation, whereas these effects are abolished after vagotomy (Siopi et al., 2023). Although chronic administration of probiotics such as *Lactobacillus rhamnosus* or *Bifidobacterium longum* reduce stress-induced corticosterone levels and ameliorate anxiety- and depression-like behaviors, these neuroregulatory benefits are absent in vagotomized animals (Bravo et al., 2011; Bercik et al., 2011). Given its dual role in inflammatory signaling and homeostatic control, the vagus nerve has emerged as a therapeutic target. Vagus nerve stimulation, which delivers electrical impulses to modulate autonomic function, has demonstrated efficacy in restoring gut microbiome balance by increasing *Lactobacillus* and *Bifidobacterium* abundance (Jung et al., 2024; Liu et al., 2024). Furthermore, vagus nerve stimulation attenuates peripheral and neural inflammation by suppressing the α7nAChR/JAK2/STAT3/NF-κB signaling axis (Chen et al., 2023). Therefore, targeted modulation of vagus nerve activity holds therapeutic promise for PND through inhibiting neuroinflammation and restoring the gut-brain crosstalk.

### 4.5 Immunity

In recent decades, substantial progress has been made in elucidating how gut microbiota regulate neuroinflammatory processes through peripheral immune mechanisms. Growing evidence indicate that surgical interventions and general anesthesia can disrupt gut microbiota homeostasis, compromise both intestinal mucosal barrier integrity and BBB function, thereby facilitating bacterial translocation and metabolite dissemination into systemic circulation (Andersson and Tracey, 2011; Minerbi and Shen, 2022). These pathophysiological alterations trigger pro-inflammatory cytokine release, enhance leukocyte chemotaxis, and promote immune cell (including macrophages, neutrophils and T cells) infiltration into the CNS (Lin et al., 2014; Saxena et al., 2021). Notably, accumulating studies suggest the gut may serve as a reservoir for brain-infiltrating immune cells during neuroinflammatory states (Schnell et al., 2021; Rustenhoven et al., 2021).

The innate immune cells such as macrophages, and neutrophils are a generic component of the organism response to infection or tissue damage, which includes surface barriers, the complement system, and inflammatory mediators produced by immune cells (Riera Romo et al., 2016). Gut dysbiosis-induced overproduction of LPS and peptidoglycans potently activates innate immune receptors, driving systemic inflammation that can breach the BBB and exacerbate neural damage (Kesika et al., 2021). This systemic inflammatory response can cross the BBB, exacerbating neuroinflammation and contributing to cognitive decline. Macrophages exhibit dual roles in orchestrating CNS responses. While essential for pathogen clearance, their surgical stress-induced CNS infiltration has been mechanistically linked to neuroinflammation, cognitive deficits, and structural brain injury (Terrando et al., 2011; Degos et al., 2013). Emerging data further demonstrate that macrophage-derived exosomes can readily penetrate the BBB, inducing hippocampal damage and cognitive impairment in PND models (Qin et al., 2024). Neutrophils constitute the first-line defense against pathogens but contribute to tissue destruction during sterile inflammation (Phillipson and Kubes, 2011; Kolaczkowska and Kubes, 2013). Chronic stress induced by surgery or anesthesia often nonspecifically activates the immune system, particularly neutrophils. This activation is characterized by an increase in neutrophil count, a decrease in lymphocyte count, and a reduction in platelet count in peripheral circulation (Alam et al., 2018; Dhabhar et al., 2012). It may involve hypercortisolism, disruption of the BBB, activation of microglia, and the release of cerebral cytokines, all of which may contribute to the pathophysiology of delirium (Hughes et al., 2012). Clinically translatable biomarkers including neutrophil-to-lymphocyte ratio (NLR) and systemic immune-inflammation index (SII) show significant correlations with PND (Wu et al., 2023; Yan et al., 2024; Zhao et al., 2023).

T cells, as adaptive immune components, constitute key players in antigen-specific immune responses. They are divided into three main functional subsets: CD8+ cytotoxic T cells, CD4+ helper T cells (Th cells), and CD4+ regulatory T cells (Tregs). Abnormal proliferation and differentiation of T cells contribute to the pathogenesis of various neuroinflammatory diseases, such as Parkinson’s disease, stroke, and autism (Kim et al., 2019, 2017; Benakis et al., 2016). Recently, the role of T cells in PND has also gained significant attention. T-cell infiltration, particularly cytotoxic T cells, has been observed during aging and in various neurodegenerative diseases, contributing to inflammation, neurogenesis inhibition, and cognitive impairment (Altendorfer et al., 2022; Stojić-Vukanić et al., 2020; Sulzer et al., 2017). Experimental evidence suggested cytotoxic T cells suppress neural stem cell proliferation via interferon-γ (IFN-γ) secretion (Li et al., 2023). Th17 cells, an important subset of CD4+ T helper cells, are crucial for mucosal immunity, particularly in the intestine, where they regulate host responses to microorganisms and maintain intestinal homeostasis (Kunkl et al., 2020; Cipollini et al., 2019). Animal studies confirmed that surgery/anesthesia -induced gut microbiota dysbiosis led to PND by regulating Th17 cell activation and IL-17 secretion (Wen et al., 2022). Conversely, Tregs critically regulate immune tolerance and suppression (Zhang et al., 2022). Tregs depletion enhanced β-amyloid clearance via choroid plexus-mediated leukocyte recruitment, while their activation supported neural recovery in stroke models (Kennedy et al., 2014; Ito et al., 2019). Current research revealed that age-related Treg functional decline exacerbated surgical stress-induced BBB dysfunction and hippocampal inflammation, whereas CD25 blockade (targeting Tregs) rescues cognitive deficits in aged murine models (Zhou et al., 2023). Gut microbiota dysbiosis exacerbates neuroinflammation by activating the innate immune cells and regulating adaptive immune cells, which ultimately drives PND.

### 4.6 Gut microbiota metabolites

The gut microbiota produces a variety of metabolites including SCFAs, neurotransmitters, secondary bile acids, and tryptophan derivatives, which influence the inflammatory response of the nervous system. Here, we summarize key mechanisms through which the gut microbiota regulates neuroinflammation via metabolic pathways.

SCFAs involving in acetate, propionate, and butyrate are the primary metabolites produced by gut microbiota through dietary fiber fermentation. These metabolites exert crucial anti-inflammatory roles both within the gut and the CNS. SCFAs can modulate neuroinflammation by regulating microglia homeostasis, inhibiting microglial activation and reducing neuroinflammatory responses (Silva et al., 2020). *In vitro*, SCFAs could suppress astrocyte activation by inhibiting NF-κB signaling (Ryu et al., 2019). SCFAs also regulated cortisol secretion by modulating the HPA axis activity and suppressed neuroinflammation by mitigating the inflammatory response (Wang et al., 2023). Notably, butyrate demonstrated additional therapeutic potential by enhancing antioxidant capacity through glutathione peroxidase upregulation and reinforcing BBB integrity via tight junction protein modulation (Heyck and Ibarra, 2019; Fock and Parnova, 2023). Collectively, SCFAs exhibit neuroinflammatory regulation through immune modulation, neuroendocrine regulation, and BBB function. The gut microbiota synthesizes neurotransmitters or their precursors, thereby modulating cerebral neurotransmitter levels and CNS functions. 5-HT derived from microbial tryptophan metabolism regulates neuronal and glial development through critical processes including cell proliferation, differentiation, and synaptogenesis (Gaspar et al., 2003). Remarkably, gut bacteria contribute over 60% of colonic and circulatory 5-HT pools, while stimulating enterochromaffin cells to release 5-HT that activates immune cells and neurons, ultimately influencing mood and cognition (Roth et al., 2021). Clinical evidence reveals that surgical/anesthesia-induced gut dysbiosis disrupts intestinal 5-HT metabolism, leading to hippocampal 5-HT deficiency and subsequent neuroinflammation (Liu et al., 2022b). Furthermore, *Lactobacillus* and *Bifidobacterium* species synthesize GABA, which mediates 20–30% of central synaptic transmission. Anesthesia impairs GABAergic signaling via p38 MAPK pathway activation, correlating with hippocampal-dependent memory deficits (Zhang et al., 2020). Emerging evidence also highlight neuroinflammatory modulation by microbial secondary bile acids and tryptophan derivatives. The intestinal bile acid pool serves as a key regulator of neuroinflammation (Mangalam et al., 2013). Microbial enzymes convert primary bile acids into secondary derivatives that enter systemic circulation, with trace amounts reaching the CNS to regulate glial functions through dedicated receptors (Mertens et al., 2017). Animal studies demonstrated that ursodeoxycholic acid supplementation alleviated oxidative stress and neuroinflammation in Parkinson’s disease models (Joo et al., 2004; Jiang et al., 2022). In addition, tryptophan-derived indoles further attenuated neuroinflammation via inhibiting pro-inflammatory cytokine production in microglia and astrocytes (Rothhammer et al., 2018, 2016). Therefore, therapeutic strategies utilizing gut microbiota metabolites become reliable treatment for PND.

## 5 Therapeutic options of targeting gut microbiota in PND

Gut microbiota dysbiosis and neuroinflammation play critical roles in the pathogenesis of PND. As discussed earlier, gut microbiota dysbiosis exacerbates neuroinflammation in PND through the gut-brain axis. Therefore, exploring the therapeutic potential of microbiota-targeted interventions to alleviate neuroinflammatory responses and improve cognitive outcomes holds significant promise for the treatment of PND. These interventions include probiotics, prebiotics, FMT, and dietary modifications, all aimed at maintaining the balance and integrity of the gut microbiota throughout the perioperative period.

### 5.1 Probiotics and prebiotics

Probiotics are live microorganisms that provide health benefits when consumed (Ng et al., 2009). They have significant potential to influence and modulate the gut microbiota. The beneficial effects of probiotics are well-documented, including improved digestion, enhanced immune function, and a reduced risk of PND (Mozaffarian et al., 2011; Tong et al., 2011; Cao et al., 2023). A meta-analysis suggested that probiotics could enhance cognitive function in individuals with mild cognitive impairment, potentially by reducing inflammatory and oxidative biomarkers (Den et al., 2020). Preclinical animal studies have also demonstrated the beneficial effects of probiotics on PND. *Lactobacillus* and *Bifidobacterium*, common components of probiotics, have been shown to protect the gut barrier and exert anti-inflammatory effects (Dalile et al., 2019). In mice undergoing anesthesia/surgery, *Lactobacillus* treatment alleviated delirium-like symptoms and neuroinflammation by improving gut microbiota imbalance and repairing synaptic loss and mitochondrial dysfunction in the brain (Liufu et al., 2020). Moreover, pretreatment of aged mice with mixed probiotics (VSL#3) mitigated anesthesia/surgery-induced memory impairment by eliminating gut microbiota dysbiosis (Jiang et al., 2019). Notably, VSL#3 treatment upregulated the expression of microRNA-146a and blocked the BTG2/Bax axis in PND mice, thereby reducing oxidative stress and neuroinflammation (Mao et al., 2021).

Prebiotics are defined as a group of substrates that can be selectively utilized by host microorganisms to modulate the gut microbiota, thereby regulating host immunity and cognition via the gut-brain axis (Chunchai et al., 2018). They are often considered complementary to probiotics or even an alternative to them. Additionally, prebiotics have shown potential in managing PND. The prebiotic Bimuno, a galactooligosaccharide (B-GOS) mixture, is a widely studied, specific nondigestible mixture designed to selectively promote the proliferation of beneficial probiotics (Vulevic et al., 2008). Early studies in humans primarily focused on the potential of prebiotics to improve gastrointestinal dysfunction in adults (Vulevic et al., 2018). More recent research have demonstrated that prebiotics can regulate brain function through the gut-brain axis. In adult rats undergoing abdominal surgery with isoflurane anesthesia, B-GOS significantly alleviated cognitive decline and reduced neuroinflammation. These effects were associated with a marked change in the β-diversity of the gut microbiome and the proliferation of Bifidobacterium and other potentially anti-inflammatory microbes (Yang et al., 2018). In both *vivo* and *vitro* models, acetate exhibited anti-neuroinflammatory activity by suppressing microglial activation, reducing the expression of inflammatory proteins, oxidative stress markers, and signaling molecules in the hippocampus (Wen et al., 2020). Together, probiotics and prebiotics may serve as potential agents for the treatment of PND. While both probiotics and prebiotics are being studied for their potential to reduce systemic and neuroinflammation associated with PND, identifying personalized probiotics or prebiotic interventions for treating PND during the perioperative period remains a crucial area requiring further investigation.

### 5.2 FMT

FMT, also known as stool transplantation or fecal bacteriotherapy, involves the transfer of a processed fecal suspension from a healthy donor into the gastrointestinal tract of a recipient. This therapeutic modality has demonstrated efficacy in treating conditions such as *Clostridium difficile* infection, inflammatory bowel disease, insulin resistance, and obesity (Xu et al., 2015; Cammarota et al., 2015; Drekonja et al., 2015). Emerging evidence further suggests its potential to ameliorate cognitive dysfunction in neurological disorders including depression, Parkinson’s disease, and Alzheimer’s disease. GF mice transplanted with fecal microbiota from patients with major depressive disorder exhibited depressive-like behaviors and metabolic dysregulation (Kelly et al., 2016). Similarly, GF mice receiving FMT from human amyloid precursor protein knock-in models developed cognitive deficits, implicating gut microbiota dysbiosis in promoting β-amyloid (Aβ) deposition and subsequent cognitive impairment (Kundu et al., 2022). Notably, in APPswe/PS1dE9 transgenic AD mice, FMT attenuated cognitive decline concurrent with reduced cerebral Aβ plaque formation and decreased tau protein hyperphosphorylation (Sun et al., 2019). These findings suggest FMT as a promising intervention for cognitive disorders. However, rigorous clinical evaluation is warranted to establish its efficacy in PND specifically associated with neuroinflammatory pathways.

### 5.3 Dietary

Dietary interventions encompasses targeted nutritional supplementation or caloric restriction to confer therapeutic benefits (Newlove-Delgado et al., 2017). Accumulating evidence indicates that specific dietary regimens may mitigate neuroinflammation. *In vitro* studies demonstrated that omega-3 fatty acids or vitamins A/D supplementation significantly attenuated LPS-induced neuroinflammatory markers in BV-2 microglial cells, with synergistic effects observed upon combinatorial administration (Kurtys et al., 2016). *In vivo*, omega-3 supplementation reversed high-fat diet-induced neuroinflammation in rats, evidenced by reduced GFAP^+^ astrocyte activation in the cerebral cortex and diminished proinflammatory cytokine levels (de Andrade et al., 2017). Furthermore, dietary restrictions have been found to improve cognitive function and mitigate neuroinflammation. Preclinical trials demonstrated that dietary restriction ameliorated PND through the modulation of gut microbiota, suppression of inflammatory responses in hippocampal microglia, and facilitation of neuronal repair and regeneration (Ren et al., 2024). Paradoxically, prolonged preoperative fasting exacerbates perioperative risks through gut dysbiosis, hypercatabolism, and stress axis activation, thereby potentiating PND pathogenesis (Kotekar et al., 2018; Batchelor et al., 2019). Consequently, tailored nutritional strategies including abbreviated fasting protocols and personalized micronutrient supplementation warrant exploration as adjunctive therapies for PND management, particularly targeting neuroinflammatory cascades.

## 6 Conclusion

In conclusion, emerging evidence demonstrate that gut microbiota plays a critical role in the neuroinflammation associated with PND through the gut-brain axis. Various perioperative factors, such as fasting, anesthesia, surgery, and the use of antibiotics or analgesics, can disrupt the diversity and composition of the gut microbiota, leading to dysbiosis. This dysbiosis triggers neuroinflammation through multiple pathways, including the disruption of the gut barrier and the BBB, HPA axis, vagus nerve signaling dysregulation, immune system activation, and metabolic alterations. Perioperative gut microbiota dysbiosis compromises the gut barrier and increases intestinal mucosal permeability, resulting in a “leaky gut”. This allows toxic microbial metabolites and other harmful substances to enter systemic circulation, activating the immune system and promoting the release of pro-inflammatory cytokines. The resulting systemic inflammation can cross the BBB, initiating neuroinflammation in the brain. This process further activates the HPA axis, compounding the inflammatory response and ultimately contributing to the development of PND. Given this evidence, preserving and restoring gut microbiota through strategies such as probiotic and prebiotic supplementation, FMT, and dietary interventions holds great promise for mitigating the onset and severity of PND. While targeting the gut microbiota to alleviate neuroinflammation has become an integral component of perioperative care, future investigations elucidating the molecular mechanisms underlying gut microbiota modulating neuroinflammation may be necessary for the discovery of novel drug targets for PND.
